# Mental Health Projects for University Students: A Systematic Review of the Scientific Literature Available in Portuguese, English, and Spanish

**DOI:** 10.3389/fsoc.2022.922017

**Published:** 2022-07-11

**Authors:** Josefina Amanda Suyo-Vega, Monica Elisa Meneses-La-Riva, Víctor Hugo Fernández-Bedoya, Ana da Costa Polonia, Angélica Inês Miotto, Sofía Almendra Alvarado-Suyo, Hitler Giovanni Ocupa-Cabrera, Maricela Alarcón-Martínez

**Affiliations:** ^1^School of Education, Universidad César Vallejo, Lima, Peru; ^2^School of Nursing, Universidad César Vallejo, Lima, Peru; ^3^School of Management, Universidad César Vallejo, Lima, Peru; ^4^School of Education, Unieuro, Federal District, Brazil; ^5^School of Audiovisual Communication and Interactive Media, Universidad Peruana de Ciencias Aplicadas, Lima, Peru

**Keywords:** mental health, college students, mental illness, intervention, health promotion

## Abstract

The mental health of college students has been the source of research, projects, and public policies involving education, health, and psychology professionals. Having as its axis the study of mental health and the phenomenon of psychological illness, this systematic review aims to characterize mental health programs directed to college students, as well as the forms of interventions offered to reduce the incidence of psychological disorders. From the proposal, a survey was conducted in the databases Scopus, Lilacs, and the repository Alicia, in the period between 2010 and 2021, choosing the search phrase “Programas de saúde mental para universitários” in Portuguese, “Mental health projects for university students” in English, and “Proyectos en salud mental para estudiantes universitarios” in Spanish. The research areas spanned humanities and social sciences, with peer-reviewed and open access articles. The questions that instigated the study were as follows: What are the mental health problems affecting college students? What type of strategy has been adopted to map the demands of university students in relation to mental illness? How can the university space reorganize itself to work on interventional-preventive aspects, according to the studies? Initially, 740 203 articles were obtained, and after sifting through 13 productions, using the PRISMA systematization. Despite several research interrelating mental health and university space, most were dedicated to data collection, using questionnaires, inventories, and scales, standardized and non-standardized. Only three studies described intervention projects and programs to reduce the problems of psychological distress in college students. Unanimously, the investigations emphasize the need for monitoring the higher education population regarding mental health and, in parallel, the implementation of institutional public policies to meet the students' demands and reduce the rates of problems in the educational field.

## Introduction

The topic of mental health in the educational space has become recurrent among the teaching community and this interest extends to education, health, and psychology professionals. The various dimensions of mental health make up an ecosystem of topics from psychoactive substance use, unplanned pregnancy, sexually transmitted infections permeating through economic health, housing, and academic-professional development (Rennó Castro, 2017; Chaves-Barboza and Rodríguez-Miranda, 2018; Marolla Garcia et al., 2020; Rangel Lucio Penha et al., 2020; Vezine Brabicoski et al., 2020; de Souza et al., 2021; Mengali and Ferraz, 2021).

In this direction, De Souza Conceição et al. (2019) highlighted the appreciable increase in mental health problems, in general, and particularly in medical students, in their systematic review. It highlights that the picture needs to be modified, due to the implications regarding health, academic-professional development, and the need to understand the factors and forms of intervention to minimize psychic suffering in academics. The complaints are characterized, mainly, Common Mental Disorder (CMD), depression, anxiety, stress, and factors that affect the quality of life of students. However, the studies did not establish the possible interactions that would allow the elaboration of systematic interventions to apprehend the phenomenon, above all, evaluating the need to increase longitudinal and qualitative studies, in order to favor the systematization of analytical models.

Expanding the discussion, Ornellas Ariño and Patta Bardagi (2018) investigated on 640 Brazilian undergraduates and analyzed the relationships between anxiety, depression, and stress with the quality of academic experiences and self-efficacy through an online questionnaire. The results strengthen the interrelations between academic space, career, and mental health problems, and the researchers reinforce the “chorus” of early identification as one of the strategies linked to minimizing psychological illness, associated with planning in the scope of higher education institutions to constitute a healthier and more positive environment. Conducting a survey on the scientific production about mental health problems in university students reverberates what has been sustained so far about the relationship between psychological illness and university activity. Especially, alerting to the imperative of recognizing, characterizing, and increasing institutional policies of mental health, through support groups and cores of specialized professionals, aware of the vulnerability of the students and actions for the prevention of injuries, favoring the completion of the course and the necessary wellbeing for the quality of the knowledge acquisition process (Alves Da Silva et al., 2021).

Given this panorama, Miranda Ribeiro et al. (2012) warn about the significant increase in drug use in the university setting, with a considerable incidence in the female population, especially amphetamines and anxiolytics. Among the various reasons listed are the use for leisure, recreation, stress relief, anxiety, and the need to break the academic routine. Contributing to the analyses, (Cuenca Robles et al., 2020) reveal that gender is one of the aspects considered in psychic illness and a factor to be incorporated into mental health projects when investigating a group of college students through a systematic literature review. Torres Sahão and Kienen (2021) dedicated themselves to understanding how the variables that may favor or hinder the adaptation of higher education students and their reflections on mental health, having as references the categories: difficulties and facilitators of adaptation, consequences of non-adaptation, symptoms, required repertoire and teaching strategies. The factors that act as barriers already widely mentioned were anxiety, stress, and depression correlated to low repertoires linked to autonomy, organization, and assumption of academic responsibilities. The researchers allude to the lack of studies that can elucidate the repertoires and reiterate the urgency of interventional processes for the population, in order to overcome this problem established in the sphere of higher education.

Chau and Vilela (2017) conducted a survey among students from Lima and Huánuco, and sought to establish a relationship between stress, avoidance style, and mental health. Their findings allowed to apprehend that mental health projects and programs focus on stress management and problem-oriented coping style, as well as socioemotional styles that have positive repercussions on the environment, behavior change, and lasting effects.

Considering the COVID-21 scenario, a significant increase in depression, anxiety, and stress has been verified as pointed out by Zapata-Ospina et al. (2021), by identifying mental health problems in 375 university students in three distinct groups: medical students, health care personnel, and the general population. The authors emphasizes the urgency for university institutions, aware of the disorders affecting students and future professionals to offer programs that focus on the mental health of academics. Andrade Gundim et al. (2021) performed an integrative literature review, in this context, pointing out that in the seven studies analyzed there is a predominance of psychological situations related to stress, grief, anger, and panic linked to academic performance, extending to the health of the student and his family, with negative repercussions on his mental health. In this direction, Son et al. (2020) implemented quali-quantitative research with 195 college students from a public educational institution with the purpose of verifying the effects of the pandemic on the mental health and wellbeing of this population, reinforcing that the pandemic negatively impacted the psychological health of students, with multiple stressors: anxiety, stress, depression, and concerns extended to family members and loved ones. Above all, the effects on the learning process that characterized concentration difficulties, reduced social interactions, concerns about academic performance, and insomnia, are essential to increase and monitor the levels of psychological illness during the pandemic.

Still, de Barros et al. (2021) in research in the Lilacs, Scielo, and Pubmed databases via Medline, obtained results analogous to those of Son et al. (2020), registering that physical and social distancing were strong contributors to the worsening condition of the mental health of college students, suggesting that studies should be carried out for the collection of accurate information, in order to investigate in more detail the problems that emerged from the pandemic.

In a multicenter study developed by Ochnik et al. (2021), involving 2,349 university students from Poland, Slovenia, Czech Republic, Russia, Germany, Turkey, Israel, and Colombia, a high prevalence of depression, anxiety, and stress among students was found, demonstrating the declining trend and worrisome data on the mental health of undergraduates in the COVID-19 setting.

It is recognized that the role of intervention on mental illness is fundamental for the maintenance and acquisition of healthy repertoires, mainly, reducing and avoiding its appearance and high incidences in the population. Scholars have affirmed and referenced the importance of maintaining mental health programs and also favoring the wellbeing of college students (Pinheiro Ramos et al., 2018; Véron et al., 2019; Hernández-Torrano et al., 2020; Rodrigues et al., 2020; Seppälä et al., 2020).

The intervention was implemented in a group of nursing students by Fernandes Garcia Severian et al. (2021), to assess the impact of a psychoeducational program on the levels of self-efficacy, self-esteem, and anxious and depressive symptoms in students at the beginning of their undergraduate studies in nursing, showed positive effects, increasing the students' feelings of self-efficacy.

Pinho (2016) also reports positive results in a program of psychological care directed to higher education students, and the complaints are similar to the framework already described: anxiety, depression, and deficits in social skills, through document analysis of medical records. Therefore, it reinforces the need for expansion and systematic offers of psycho-pedagogical support to reduce mental disorders and also to trigger preventive factors in the higher education scenario. Rangel Lucio Penha et al. (2020) point out the need to broaden the spectrum of psychological health support and include graduate students, as they share the same problems of psychological illness and, sometimes, do not receive the institutional psycho-pedagogical, psychological, and psychiatric support that is offered to undergraduate students.

Reinforcing the above guidelines, Barbosa Rozeira et al. (2018) detailed the importance of support services for students, mainly, in developing repertoire to manage the problems arising from academic life, as well as, those of personal origin that affect academic development, and, in addition, fostering health actions employing group activities. Thus, favoring health, the achievement of their goals, satisfaction, academic performance, and educational success (Soares Saraiva and Aredes Almeida, 2019; Pinheiro-Carozzo et al., 2020). Due to this scenario, the high incidence of psychological illness in college students (Macêdo et al., 2021) reverberates the urgency of the implementation of support services to meet the psychopedagogical demand of college students and that such services are the focus of public policies in health and also institutional, as reiterated by Santomauro et al. (2021).

Having as the axis of the study of mental health and the phenomenon of psychological illness, this systematic review, aims to characterize mental health programs directed to college students, as well as, forms of intervention offered to reduce the incidence of psychological disorders. Thus, it asks: What are the mental health problems that affect college students? What kind of strategy has been adopted to map the demands of university students in relation to mental illness? How can the university space reorganize itself to work on interventive-preventive aspects, according to the studies?

## Materials and Methods

This research is characterized by a systematic literature review defined as a research methodology that adopts rigorous criteria on the collection of studies related to a particular field of knowledge or phenomenon. In this sense, it seeks to evaluate the quality of the studies, especially, their applicability in the scenario so that they can implement transformations (Ornellas Ariño and Patta Bardagi, 2018; Camilo Rosas et al., 2021).

It is essential to organize a protocol that describes the process and the parameters adopted so that it is replicable, under similar conditions, by other scholars.

Complementing the discussion, Dos Passos Canteri et al. (2019); Camilo Rosas et al. (2021); de Souza et al. (2021) point out an important tool to assist, having as reference the Preferred Reporting Items for Systematic Reviews and Meta-Analyses, widely recognized as PRISMA, which systematizes a set of items essential for conducting a systematic review (PRISMA checklist), by explaining the inclusion and exclusion criteria that guide a given systematic review set up (PRISMA flow diagram) of a visual nature (Liberati et al., 2009; Moher et al., 2009). They also indicate the CASP (Systematic Review Checklist), by listing a list of questions that seek to reflect and analyze the results obtained and, consequently, the validity, and enabling the analysis of what would be feasible, from the results, for application in a given context.

The PRISMA flow diagram was used in the research. It can indicate the number of articles identified, screened, eligible, and included in each phase.

Another crucial point for the systematic literature review is the selection of the database, which must consider the area or areas of knowledge that one wishes to investigate (Kitchenham, 2004). In this regard, search strategies are one of the essential factors in the collection of articles. Being essential, the domain of structures is linked to the choice of words, synonyms, phrases, and even technological competence for the Boolean search (Pereira and Diniz Júnior, 2017).

The search was conducted in three databases: Scopus, Alicia, and Lilacs. Among the databases consulted Scopus is considered one of the most important for scientific consultations, with reputable journals and benchmarked by peers, composed of abstracts, citations, books, proceedings of events from various areas of knowledge (de C. S. Casarin and de Paulo, 2020). The journals available at the library of Concytec (National Council for Science, Technology and Technological Innovation) in the national repository Alicia, bring together Peruvian productions and also aggregates the Senescyt database (Secretariat of Higher Education, Science, Technology and Innovation) for access to Ecuadorian products (Barrutia Barreto et al., 2021). Extending the collection process, consultation was also performed in Lilacs (Latin American Literature on Health Sciences), which compiles journals, dissertations, and theses on health science (Laerte Packer et al., 2007).

It is important to emphasize that phrases were used to search for scientific evidence. Initially, when conducting a search with keywords and Boolean operators, a large number of articles were found, but with information irrelevant to the research. It was necessary to make a decision and search with exact phrases in the three databases in the three languages, this search strategy follows the guidelines of (Vera Carrasco, 2009).

**Figure 1:**
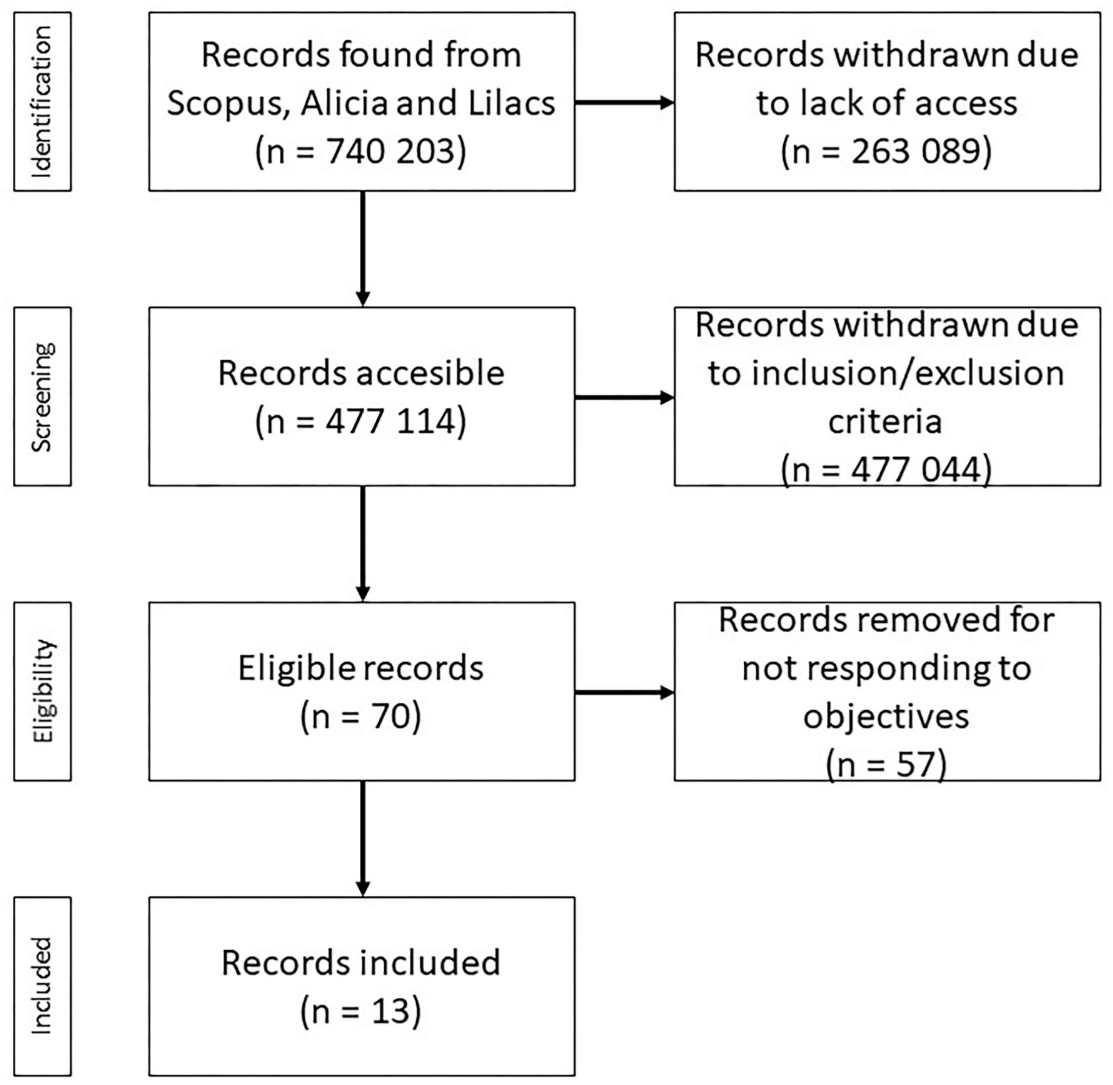
PRISMA flow chart.

For the selection of articles, in line with the objectives of the systematic review, the following inclusion criteria were considered: (a) period 2010-2021; (b) publication of researchers from South America; (c) search phrases in Portuguese, English, and Spanish; (d) empirical articles and experience report with open access; (e) articles with peer review; (f) target audience: undergraduate students; (g) research areas: human and social sciences; and (h) concordance with the research objectives and topic of study. And among the exclusion criteria: (a) monographs, dissertations, theses, and E-books; (b) opinion articles and editorials; (c) review articles. The search phrase programas de saúde mental para universitários' was translated into English ‘Mental health projects for university students' and Spanish ‘Proyectos en salud mental para estudiantes universitarios'. The search results are detailed in Table 1 and the detailed process in Figure 1.

**Table 1:** Database, search (Portuguese, English, and Spanish language) and article inclusion criteria by phase.

**Database**	**Search (Portuguese, English, and Spanish)**	**First phase**	**Second phase**	**Third phase**
Scopus	Programas em saúde mental para universitários	0	0	0
Scopus	Mental health projects for university students	19,191	1	0
Scopus	Proyectos en salud mental para estudiantes universitarios	0	0	0
Alicia	Programas em saúde mental para universitários	2,696	6	1
Alicia	Mental health projects for university students	708,032	2	0
Alicia	Proyectos en salud mental para estudiantes universitarios	8,917	15	0
Lilacs	Programas em saúde mental para universitários	780	39	11
Lilacs	Mental health projects for university students	442	3	0
Lilacs	Proyectos en salud mental para estudiantes universitarios	146	6	1
**Total**		**740,203**	**70**	**13**

## Results

After using the filter to select articles, out of a total of 740,203, 70 were found in the second stage and 13 in the third stage. These 13 allowed the construction of items that resulted in three analysis matrices, with the following indicators: (1) author, year, country, the purpose of the proposal, participants (Table 2); (2) approach, instruments employed, and type of intervention (Table 3); and (3) results, conclusions and recommendations (Table 4) that will guide the analyses and discussion.

**Table 2:** Identification: author, year, country, objective, and participants.

**Code**	**Author, year, country**	**Objective**	**Participants**
1	(Pereira Prates et al., 2012) (Brazil, 2012)	To determine the lifestyle indicators associated with negative self-assessment of health in Physical Education students.	217 Physical Education students (54.8% male) from a public institution in the Northeast of Brazil of Brazil.
2	(Silva de Souza et al., 2010) (Brazil, 2010)	To assess the relationship between family support, mental health, and risk behaviors.	766 university students (average age 21.53).
3	(Alves Da Silva et al., 2021) (Brazil, 2021)	To identify the prevalence, severity, and factors associated with depression among college students in the Federal District (Brazil).	Cross-sectional study of descriptive character, with 521 students from a private institution
4	(Pinheiro Ramos et al., 2018) (Brazil, 2018)	To present a set of interventions carried out in a Brazilian public university, focusing on dimensions related to career planning, development of study habits, and promotion of the mental health of university students.	86 psychology undergraduates (conducting the activities) and that benefited 705 university students (attended).
5	(Pires et al., 2020) (Brazil, 2020)	To assess the pattern of alcohol and other psychoactive substance use in college students.	180 psychology undergraduates (age ranging from 17 to 42 years).
6	(Campos Osse and Izídio da Costa, 2011) (Brazil, 2011)	To map the psychosocial conditions and quality of life of university students in the student housing of the University of Brasilia.	87 volunteers with an average age of twenty-two (public university).
7	(Murakami et al., 2018) (Brazil, 2018)	To describe how CAEP works, recalling its history and presenting its experiences and perspectives for interventions in the assistance to students, and advice to teachers and courses.	Not applicable, historization of the development process and psychological and psycho-pedagogical services offered.
8	(Dal Maso and Biasotto Feitosa, 2013) (Brazil, 2013)	To assess neuroticism in university students in the state of Rondônia.	1,031 university students of both genders, between 18 and 75 years old.
9	(do Nascimento Lameu et al., 2015) (Brazil, 2016)	To assess the prevalence of stress symptoms among undergraduate students.	635 students from the Federal Rural University of Rio de Janeiro.
10	(da Cunha et al., 2012) (Brazil, 2012)	To investigate the relationship between alcohol abuse or dependence and the occurrence of impairment in social skills in a sample of college students.	113 participants, aged between 18 and 53 years, and university students from Porto Alegre (RS) and Metropolitan Region, convenience sample.
11	(Bacha et al., 2012) (Brazil, 2012)	To evaluate the quality of life of psychology students and correlate it with sociodemographic factors.	310 psychology students.
12	(Rivadeneira Guerrero et al., 2020) (Ecuador, 2020)	To train a group of undergraduate students as university health promoters and evaluate the results.	A total of 148 involved: Capacitation of an interdisciplinary group composed of 13 students in the period 2012–2014 (multiplier-intervention group); 120 undergraduate students from various fields of knowledge, from all semesters (course participants); 15 undergraduate and graduate professors for the development of the themes in mental health.
13	(Castro-Silva et al., 2021) (Brazil, 2021)	To identify the presence of burnout and its subscales in Psychology students	135 academics from the 1st, 3rd, 5th, 7th, and 9th semesters.

**Table 3:** Approach, instrument and type of intervention.

**Code**	**Approach**	**Instrument**	**Type of intervention**
1	Cross-sectional study of a quantitative nature.	Questionnaires (self-assessment of health, socioeconomic, demographic and lifestyle conditions).	Survey by means of a questionnaire.
2	Quantitative.	-Family Support Perception Inventory (IPSF); - Self Reporting Questionnaire (SRQ-20); - Student Behavior Identification Questionnaire (QICE).	Survey using two types of questionnaires.
3	Quantitative.	Patient Health Questionnaire-9 (PHQ-9).	Survey by means of a scale.
4	Mixed.	Semi-open questionnaire for each of the projects.	-Connected Extension Projects: 1. reception and psychological screening; 2. workshops: preparation for academic life, social skills, anxiety control and coping with stress, study orientation, and specific themes; 3. individual psychotherapy; 4. education for career Semi-open questionnaires.
5	Cross-sectional descriptive study, quantitative nature.	-Questionnaire: Alcohol Use Disorder Identification Test – (AUDIT). Smoking and Substance Involvement Screening Test (ASSIST).	Survey by means of two instruments.
6	Quantitative.	- Childhood Abuse and Trauma Scale (CAT); - Life Experiences Survey (LES); - Suicidal Behavior Questionnaire (SBQ-R); - Positive and Negative Suicide Ideation (PANSI)-translated and adapted for the university group; - Alcohol Smoking and Substance Involvement Screening Test (ASSIST) was adapted for the study; - Minnesota Multiphasic Personality Inventory 2 (MMPI-2) adapted for university students.	Survey by means of a questionnaire employing six scales.
7	Qualitative (survey of the services offered by CAPE).	Records from 2014–2017: attendance for 655 students, generating 3 996 attendances.	Intervention-prevention actions: - Three pillars of action: teaching (advising the Undergraduate Committee and the Faculty's faculty), research (developing projects and research on education in the health professions, pedagogical, psychosocial and psychopedagogical characterization of its student population) and assistance (developing psychopedagogical and psychological support activities for undergraduate students) - Service to students: (a) psychological screening, (b) brief psychotherapy, (c) educational orientation (psycho-pedagogical and/or pedagogical).
8	Quantitative: survey research, with sampling from a population of interest for later descriptive and inferential statistical analysis.	- Factorial Scale of Neuroticism (EFN); - Form with sociodemographic items (elaborated from the Criterion for Economic Classification Brazil-CCEB.	Application of neuroticism scale and sociodemographic form.
9	Quantitative.	- Lipp's inventory of symptoms of stress for adults (ISSL); - Questionnaire.	Survey using two instruments.
10	Quantitative.	-Alcohol Use Disorders Identification Test (AUDIT) –WHO (translated into Portuguese by Figlie, Pillon, Laranjeira, and Dunn, 1997 and validated for use in the Brazilian population. - Inventário de Habilidades Sociais (IHS).	Survey using two instruments.
11	Quantitative.	-Sociodemographic questionnaire - The Medical Outcomes Study 36-item Short-Form Health Survey (SF-36): to evaluate the quality of life.	Survey using two instruments.
12	Mixed: Participatory Action Research.	-Satisfaction questionnaire for project participants and the result of discourse analysis: perception and satisfaction with the support network and discourse analysis about the activities and impact of the network; - Questionnaire: perception and satisfaction with the support network; - Semistructured interview (focus group): investigating problems in academic and university spaces, health, emotional, family and social.	Prevention-intervention actions: - Steps: (1) training of the group of 13 students as multipliers in health promotion; (2) diagnostics of the students' general health situation (*n =* 120 students); (3) outlining, planning and development of the project by the trained students; and (4) evaluation of the intervention (*n =* 120) - Duration of the project: 13 meetings, 14 theoretical-practical workshops, 5 seminars in the institutional space and 4 field visits in a rural area (552 face-to-face hours, March 8 to December 22, 2013).
13	Cross-sectional descriptive study of a quantitative nature.	-Demographic questionnaire; - Maslach Burnout Inventory Student Survey (MBI-SS) validated for Portuguese.	Survey using two instruments.

**Table 4:** Results, conclusions and recommendations.

**Code**	**Results**	**Conclusions**	**Recommendations**
1	- Reported problems: inadequate diet, restricted hours of sleep, habit of not using seat belts, unprotected sexual activity, and stress; - Inadequate behavior: anger, hostility, bad mood, nonplanning of tasks; - Problems with introspection: demotivation, depression, sadness, pessimism.	- Negative styles and negative health evaluation: inappropriate eating habits, sleep problems, not using seat belts, stress, unprotected sex, type of inappropriate behavior, and introspection problems; - Contributions of the study: few investigations on the subject in the field of PE; questionnaire that investigates multiple areas of health; probabilistic sample.	- Developing strategies to foster interaction among students, educational programs focused on healthy lifestyles and health promotion: safe sex, seat belt use, social network support.
2	- Confirmed the relationship between the variables family support, mental health and risk behaviors.	- Female gender: higher rate of mental disorders due to multitasking situation, in addition to gender discrimination, violence, selfimage (eating disorders) - Family support and risk behavior are associated; - correlation between family support, mental health and eating habits	- Identification of risk factors to design prevention and intervention programs for health care and mental health. - Perception of care is closely linked to family support, and also to the development of healthy habits.
3	- Depressive symptoms were 521 (96.6%) college students, of which 31.3% had mild depression, 23.4%, minimal depression, 13.1% moderately severe depression, 9.6% severe depression, and 9.2% moderate depression. - A correlation was found between family income and semester attended.	- Confirmation of depressive symptoms in college students ranging from mild to severe.	- Necessity of identifying conditions that favor depression and, concomitantly, fostering mental health programs.
4	- Good performance of Psychology students when implementing psychotherapeutic care; - Importance of the themes approached in the projects; - Support in the psychological consultations.	- Intervention regarding the adaptation to university life and, in parallel, experiences under supervision of Psychology students (internship) with strengthening of the construction of a professional identity.	- Limitations: need for systematic evaluation of the interventions made. - Implement actions based on the national student assistance policy: psychological attention to the university student to reduce illness, absenteeism, adaptation to academic life, and promotion of mental health. - Promote the articulation between extension, research, and teaching.
5	- Prevalence of alcohol use: 81.7% lifetime, 67.6% in the past three months, and 55% in the binge pattern (indicative of an alcohol level of 0.08d/l or five doses for men and four for women).	- Justifications for alcohol use: enhancer of the desire to smoke, followed by its perception as a gregarious factor at parties/social gatherings and as a facilitator of stress coping - Risk behaviors: drunk driving, unprotected sexual activity, conflict and disorder as legal problems. - Higher exposure of college students due to stress, permissive environment, pleasures and sensations when consuming SPA (psychoactive substances).	- Prevention programs and public policies guided by the notions of selfcare, protagonism, and active participation in one's own rehabilitation - Increase and popularization of licit and illicit drugs among college students. - Most commonly used drugs: alcohol, tobacco, and marijuana, identify the forms of consumption and the scenarios; - Recent licit and illicit drug use in the population under study.
6	- Scenarios: students dependent on institutional resources, beginning their course, living in cities outside Brasilia - Anxiety, depression, emotional instability and barriers to institutional support.	- In addition to institutional care, there is a need for psychotherapeutic support; - Negative childhood experience: neglect, unfavorable family environment due to physical and sexual abuse; - Alcohol use: ranging from regular to sporadic; - Situations of anxiety, depression, associated with resistance to ask for and accept help were reported.	- The assistance programs are inefficient to meet the students' demands, thus, they must be expanded, considering the emerging needs. - Necessity of emergency actions and institution of new services to support permanence and quality of life; - Anxiety traits varying among the group and as for suicidal ideation, the results were average, indicating low risk, due to the predominance of positive thoughts; - Monitoring and support to students at the beginning of their course, due to adaptation, stress and initial difficulties.
7	- Queixas: ansiedade, depress ao sintomas de depress ao, transtorno de ajustamento, dificuldades de relacionamento interpessoal, problemas acadêmicos e apoio vocacional.	- Higher education institutions must know the contexts and demands of students in order to elaborate and increase institutional support strategies for the complexity and multiplicity of demands in higher education. - Identify the psychosocial demands of students that affect their insertion, quality of life, and learning process.	- Foster and strengthen the different student support services, directly or indirectly, to continue studies, promote mental health and academic success.
8	- Women indicated a higher degree than men on the neuroticism scale; - Men were more prone to social maladjustment, especially in singles; - Depressive symptoms reported in the female audience.	- Women have a greater tendency to present psychological suffering than men; - Male depression is still understudied; - Neuroticism varied considering gender and marital status; - Relationship between neuroticism and mental health.	- Study neuroticism and its reflection on the mental health of college students for psychological or psychopedagogical interventions; - To characterize the forms of depression that are reported by men; - To study neuroticism and its reflection in the mental health of university students for psychological or psychopedagogical interventions; - To characterize the forms of depression reported by men.
9	- Half of the sample was indicative of stress - Highest level of stress among females, - Lower levels of stress in students who live with their families - Students who have shared living arrangements, in public spaces, report being more stressed.	- Gender differences in stress are explained by the multiplicity of activities taken on by women. - Family support and proximity are factors that can help mental health - Living in public spaces implies living with people from different cultures and habits, and in sororities, due to rotation and parties, there are fewer opportunities to relax and concentrate on studies.	- Higher education institutions should be concerned with the adaptive processes that students go through that are stressgenerating and significantly affect mental health. - Offering and guidance from psychology and psychiatry services are important and should exist in the educational space.
10	- Alta incidência do uso de álcool em universitários (beber problemático) e padr ao binge de consumo, sem diferença entre os géneros.	- Risk behaviors: drunk driving, fighting, unprotected sex, and association with other drugs; - Record of ‘blackouts' and possible addiction, as well as being a danger to people around; - Interrelationships between alcoholism and deficits in social skills; - Limitations: age variability.	- The need for policies directed to the health of the university population; - Development of a program for social skills in college students: evaluation and promotion; - Close interaction between social skills and professional competences; - Creation of mental health support groups, especially for alcohol abuse, since declining interaction skills can be a consequence of alcoholism. - Suggestion: studies involving pre and post intervention followup with a control group.
11	- The academics showed higher scores in “functional capacity” (mean 87.43) and lower scores in “vitality” (mean 58.50). - Gender differences: in pain domain, women obtained lower scores. - The relationship between family income, pain, vitality, social aspects and mental health was a relevant aspect.	- The total score of quality of life is worse in the first year and its quality increases as the course goes on; - The quality of life index was satisfactory, however, there is a need to offer programs to support the mental health of university students.	- Recognize that those entering undergraduate courses may have support and welcoming from the educational institution, with quality of life and mental health programs; - Training to favor healthy repertoires.
12	- Training of health leaders: development of skills for decision making and confidence building with the following products: empowerment, listening process, participation, and followup that are fundamental for community work; - Addressing health issues is essential; - Evaluation of the impact of the networking activity: novice students 76.2% (dealing with problems), 90.6% (personal, spiritual, affective and physical development), 94% (interpersonal relationships improved), 68.9% (knowledge about their rights) and 38% (changed their habits of life).	- There is an imperative to strengthen the educational processes directed to health promotion in undergraduate courses; - The project's contribution is differentiated by the use of an interactive methodology, empowering and multiplying nature, where students are protagonists and develop activities with their peers; - Networked projects must involve all courses emphasizing promotion, prevention, not being restricted to specific campaigns, lectures, without moving from individual perceptions to collective ones.	- Avoid carrying out specific activities on health promotion, such as on specific dates, and replace them with programs and projects during undergraduate training; - To employ interactive and participatory methodologies, as well as strategies involving actionresearch, aiming at changing behaviors.
13	- No gender differences were found regarding Burnout; - In the sample, 20% presented the syndrome; - The third and fifth periods had a higher incidence of Burnout than the others.	- Importance of characterizing Burnout in students to perform preventive interventions, minimizing its negative effects during the course and in future professional occupation. - To outline pedagogical strategies or programs that aim at the improvement of social skills, as a form of intervention; - To propose complementary activities that have as their axis the prevention of Burnout in university students.	- Institution of monitoring the mental health of students during their university education. - To implement online spaces for the diffusion of therapeutic support, such as cognitive behavioral therapy, meditation, mindfulness, breathing and relaxation as a way to reduce stress. - Implement institutional policies of welcoming, such as those developed by psychosocial centers, with a view to mental health, serving students who depend on the university space housing (promotion of wellbeing and comfort).

## Discussion

One of the most revealing findings was to find 12 scientific evidence in Portuguese, 1 in Spanish, and 0 in English. The vast majority of scientific articles came from the Lilacs database (Literatura Latinoamericana y del Caribe en Ciencias de la Salud; Latin American and Caribbean Health Sciences Literature). This shows that there is a wide scientific production specialized in health that does not achieve visibility due to language or its publication in journals that are not indexed in popular databases.

When checking the publications considering the South American countries, the following distribution is observed: Brazil (*n* = 12) and Ecuador (*n* = 1). South America is composed of 13 countries and only two, in the period 2010-2021, published on the subject in the Scopus, Lilacs, and Alicia databases. This is a worrisome picture, since mental health problems have been increasing in the university population, and it is sine qua non to promote investigations, prevention, and intervention programs, aimed at increasing support to students. Admittedly, these students will be future professionals who will be working effectively with the population. Consubstantiating this scenario, Barrutia Barreto et al. (2021) assert that countries such as Peru and Ecuador have low scientific production and urgently need to invest to change this picture.

Focusing on the objectives of the studies, in general, the research sought to associate mental health with the following factors: quality of life, family support and risk behavior, career planning, and study habits, housing conditions offered by the educational institution, and psychosocial factors of the students, alcohol use and deficits in the field of social skills and the already known themes such as depression, use of psychoactive substances (licit and illicit), burnout and stress.

Regarding the methodology employed, it is important to emphasize that only 1 of the scientific evidence identified employed documentary analysis (Murakami et al., 2018), this article was conducted in Brazil and written in Portuguese.

As for the remaining 12 pieces of scientific evidence, all were non-experimental studies in which university students were used as a sample, with sample sizes ranging from a minimum of 86 (Pinheiro Ramos et al., 2018) to a maximum of 1,031 (Dal Maso and Biasotto Feitosa, 2013).

It should be noted that of the 13 productions, only 2 studies addressed and described health promotion programs and intervention dynamics, the others invested in the application of standardized and non-standardized instruments for data collection. The quantitative (*n* = 10), mixed (*n* = 2) and qualitative (*n* = 1) approaches predominated. The use of instruments, including sociodemographic questionnaire, totaled 11 articles, thus, with the use of two (Bacha et al., 2012; da Cunha et al., 2012; Dal Maso and Biasotto Feitosa, 2013; do Nascimento Lameu et al., 2015; Pires et al., 2020; Castro-Silva et al., 2021) and six instruments (Campos Osse and Izídio da Costa, 2011). However, four studies designed their own instruments for data collection (Pereira Prates et al., 2012), and one used document analysis (Murakami et al., 2018) to capture the information. It is still observed the trend of using a single instrument to implement the collection of information, however, it would be interesting to combine two or more to capture the complexity and dynamics of the phenomenon, in this case, of mental health. As identified, one study combined questionnaires and semi-structured interviews, involving two participants: teachers and students.

However, the two studies developed health promotion actions with interventions (Pinheiro Ramos et al., 2018) provided by the experience in the internship in psychology, in which students were mental health agents, attended by 705 college students. A similar program has been developed by Rivadeneira Guerrero et al. (2020), who also worked with students with health multiplying agents and empowered them for network actions. This last study, also involved the expertise of teachers supporting the workshops as to the contents and specific themes addressed. Those who reported the experience of health promotion highlight the intervening factors and reiterate the good performance of Psychology students and their future preparation to work with the community, concomitant, and the supervision of the attendances (Pinheiro Ramos et al., 2018). Similar results were pointed out by the research of Rivadeneira Guerrero et al. (2020), describing the benefits of training an interdisciplinary group of students for interventions and acting with the coethnics of higher education courses. Emphasizing empowerment, the ability to listen to others, and the awareness of community work directed to mental health prevention. The records of the students who participated in the preventive actions underlined the development in the personal, affective, and physical areas, providing quality social interactions and a change in habits after the intervention.

The results of the investigations are similar regarding the complaints in relation to the mental health situation, in this way, regarding risk behavior, the authors highlighted inadequate diet (Silva de Souza et al., 2010; Santos Silva, 2012), restricted hours of sleep, the habit of not using seat belts (Pereira Prates et al., 2012), excessive alcohol consumption (da Cunha et al., 2012; Miranda Ribeiro et al., 2012), leading users to get involved more frequently in fights and conflicts with the law, in addition to unprotected sexual activity, drunk driving endangering their lives and those with them. Considering gender and the incidence of mental problems and disorders, task overload, sexism, violence against women, body dysmorphia, expressed by eating disorders_ anorexia, and bulimia (Silva de Souza et al., 2010) are registered as possible vectors. There was the characterization of depressive symptoms of different degrees in the university public (Dal Maso and Biasotto Feitosa, 2013), due to student housing (in the university environment) and family distance, as well as dependence on institutional resources to continue studies, such as scholarships and other forms of support, generating anxiety, emotional instability and discredit as to the support offered (Campos Osse and Izídio da Costa, 2011). In one study, Murakami et al. (2018) also characterized adjustment disorders, conflicts of interpersonal nature, academic problems and the need for vocational support, as significantly affecting the quality of life (Bacha et al., 2012) and the interaction between the lifestyles adopted, factors such as stress (do Nascimento Lameu et al., 2015) and Burnout syndrome in academics (Castro-Silva et al., 2021), resulting in absenteeism, dropout and little investment in studies. Moreover, the manifestation of internalized (demotivation, sadness, pessimism) and externalized (fights, hostility) behaviors, is associated with the non-planning of tasks (Pereira Prates et al., 2012).

**Figure 2:**
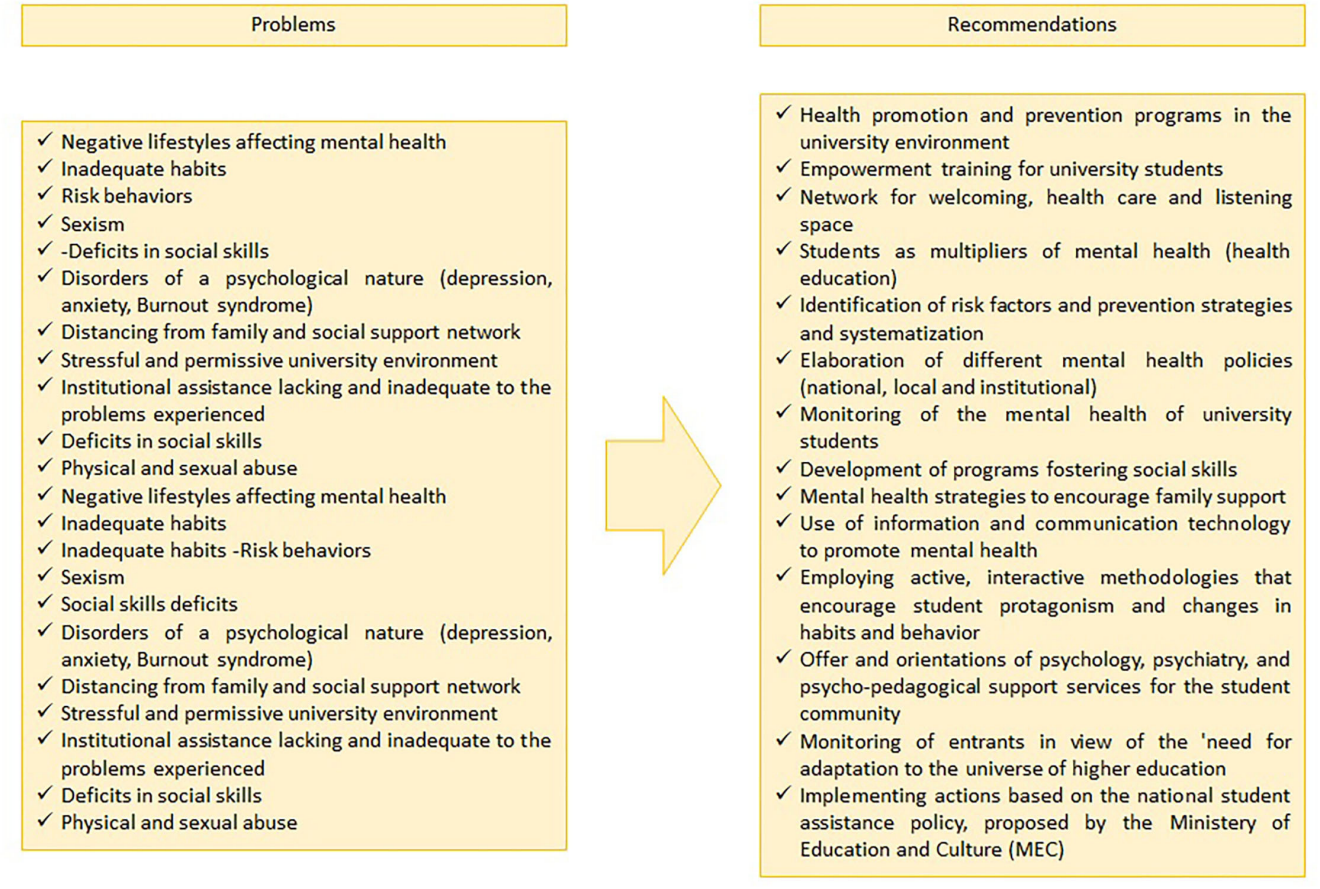
Problems and recommendations identified.

The findings reinforce the problems found in the studies regarding the mental health of the university community (Figure 2) and identified by the selected instruments: (a) risk behavior and inappropriate habits for physical and mental health (Campos Osse and Izídio da Costa, 2011; da Cunha et al., 2012; Pereira Prates et al., 2012; Pires et al., 2020); (b) disorders of psychological origin: depression, stress, Burnout syndrome (Campos Osse and Izídio da Costa, 2011; Bacha et al., 2012; Pires et al., 2020; Andrade Gundim et al., 2021; Castro-Silva et al., 2021); (c) cultural factors related to gender (Silva de Souza et al., 2010; Dal Maso and Biasotto Feitosa, 2013; do Nascimento Lameu et al., 2015); (d) stressful university scenario: from housing, going through semesters attended to collective and individual demands (Campos Osse and Izídio da Costa, 2011; do Nascimento Lameu et al., 2015); (e) little knowledge of the psychosocial demands and needs of university students by higher education institutions (Murakami et al., 2018); (f) absence or little investment in prevention and health promotion programs for network actions (Bacha et al., 2012; Rivadeneira Guerrero et al., 2020); (g) scarce studies on the subject in higher education (Pereira Prates et al., 2012); (h) conflicts and absence of family support (Silva de Souza et al., 2010; Campos Osse and Izídio da Costa, 2011; do Nascimento Lameu et al., 2015); and (h) deficits in social skills (Campos Osse and Izídio da Costa, 2011; da Cunha et al., 2012).

It is unanimous the defense of programs and groups directed to the mental health of university students, by the authors of the articles, and emphasize that the proposals should not be restricted to punctual moments or calendar events. The continuity of programs and systematization of the welcoming, guidance, and monitoring of students must be subsidized by health and education policies, based on and solidified by national guidelines, and also by the mapping of needs, interests, and shortages identified by each institution of higher education. Moreover, the formation of multipliers among students is a goal to be achieved, to the extent that they will be future professionals and can spread the promotion with their classmates, groups, at work, and among those with whom they maintain interpersonal relationships.

## Conclusion

The concern with the mental health of the student community in higher education has mobilized researchers, educators, and specialists who develop public policies. Despite the alarming picture, these actions are still timid and need to be expanded in the university context. This systematic review pointed out that many investigations are using a diversity of instruments, however, the translation of the results needs to be actioned and feed to the projects, programs, or groups that work with mental health. Only three studies reported the experience of a program, one contextualizing and performing the historicization of mental health care and two others, describing the intervention scenario, envisioning the students as multipliers and effectively being empowered. One sets up the activation of the support network with teachers and students together building an intervention-prevention methodology. This collaborative posture reaffirms that teachers, students, and employees of the educational institution are effectively health promoters, by virtue of being in direct and constant interactions. Mainly, it breaks the idea that mental health is a topic outside the classroom and educational research institutes.

When studying a multifaceted phenomenon such as mental health, it is fundamental to know the peculiarities, needs, and claims that are established on an individual and collective level, as well as to seek, in the research architecture, selective instruments to collect information, allowing the triangulation of information and avoiding focusing on only one source of information. As already referenced, the monitoring of situations involving mental health risks and the development of social skills are differential elements. De Souza Conceição et al. (2019) proposed a systematic approach for studies involving mental health, expanding the implementation of longitudinal and qualitative studies to systematize models that can contribute to the development of health promotion and prevention programs in higher education. Therefore, the diffusion of data to the internal and external community is another sensitive point to be investigated. It is well known that young students will enter the labor market at the end of their education, improving their community through their work.

Reflecting on the limitations of this study, it is important to point out that initially, the search was performed using a search equation combining the Booleans OR and AND, which did not prove to be fruitful, and the option for the search phrase proved to be more appropriate for compiling the articles in the databases. We encourage other researchers in the field to search both Booleans and phrases for future systematic reviews to identify articles that were not found in this study and to generate academic discussion.

Likewise, it is important to emphasize that the Scopus database was accessed through the credentials granted to the research group through their home institutions. Since we are based in South America, the vast majority of scientific articles with full download access are those written in Portuguese and Spanish.

The geographical area was selected due to the proximity of the university scenarios and similarities in mental health problems involving health policies and governmental investment in higher education. It is believed that other European countries as well as the United States have well-differentiated policies regarding the reception and mental health projects for the university population, as well as lines of investment and differentiated care by specialized teams. Overall, it was observed that some articles were dedicated to investigating mental health problems, especially by surveying the problems found in the group of college students, only three actually proposed intervention-prevention actions, and a fourth the functioning of a mental health support center. However, the results of the 13 studies make it possible to obtain indicators of the main problems and the development of interventional and baseline strategies for the elaboration of mental health prevention programs for college students.

The psychometric properties of the instruments in the articles were not analyzed as they were not the object of the investigation but were presented, as they were validated and standardized nationally and internationally for mental health research, and others were developed (e.g., questionnaires - not standardized) to meet the objectives of the study and make data collection possible.

Further future studies are necessary to evaluate some points: first, to also search for experience reports that can expand the collection of articles about mental health programs and, concomitantly, the forms of intervention adopted. Second, the filter that delimited the selection encompassing the area of human and social sciences should be extended to the health area; and third, the Web of Science database should be included due to the flow of articles and the quality of the journals.

## Data Availability Statement

The raw data supporting the conclusions of this article will be made available by the authors, without undue reservation.

## Author Contributions

All authors listed have made a substantial, direct, and intellectual contribution to the work and approved it for publication.

## Funding

This study was carried out and funded by the Universidad César Vallejo, within the framework of the work plan outlined in RVI N° 052-2019-VI-UCV.

## Conflict of Interest

The authors declare that the research was conducted in the absence of any commercial or financial relationships that could be construed as a potential conflict of interest.

## Publisher's Note

All claims expressed in this article are solely those of the authors and do not necessarily represent those of their affiliated organizations, or those of the publisher, the editors and the reviewers. Any product that may be evaluated in this article, or claim that may be made by its manufacturer, is not guaranteed or endorsed by the publisher.
